# Performance of the Heaviness of Smoking Index in Indian Settings

**DOI:** 10.7759/cureus.50433

**Published:** 2023-12-13

**Authors:** Sagarika Das, Pratap K Jena, Nancy Satpathy, Jugal Kishore, Kavitha AK, Venkatarao Epari, Rashmirekha Gadtia

**Affiliations:** 1 Nursing, Shri Jagdishprasad Jhabarmal Tibrewala (JJT) University, Jhunjhunu, IND; 2 Mental Health Nursing, Central Institute of Psychiatry, Ranchi, IND; 3 Health Care Management, Swiss School of Business and Management (SSBM) Geneva, Geneva, CHE; 4 Public Health, School of Public Health, Kalinga Institute of Industrial Technology (KIIT), Bhubaneswar, IND; 5 Community Medicine, Institute of Medical Sciences and SUM Hospital, Siksha 'O' Anusandhan, Deemed to be University, Bhubaneswar, IND; 6 Community Medicine, Vardhman Mahavir Medical College and Safdarjung Hospital, New Delhi, IND; 7 Public Health, Indian Medical Council of Medical Research (ICMR) Regional Medical Research Centre, Bhubaneswar, IND; 8 Epidemiology, National Health Mission, Madhya Pradesh, Harda, IND

**Keywords:** global adult tobacco surveys, tobacco use assessment, quit attempts, tobacco consumption patterns, heaviness of smoking index, nicotine dependence measurement

## Abstract

Background and objective

The heaviness of smoking index (HSI) is a popular tool to assess nicotine dependence in clinical and community settings. Low cigarette consumption and concurrent use of other tobacco products raise concerns about its validity in Indian settings. This study explores the performance of HSI in Indian settings.

Methods

This study analyzed daily manufactured cigarette smoker data from the cross-sectional Global Adult Tobacco Surveys (GATS) from its first (GAST-1, 2009) and second waves (GATS-2, 2016), both of which were available in the public domain. The HSI scores were calculated based on the number of cigarettes smoked per day (CPD) and time to first smoke (TTFS) after waking up among the current daily cigarette users. This study examined the utility of the HSI scale in Indian settings by estimating the predictability of low dependence on quit attempts and quit intentions using the likelihood ratio parameter.

Results

About nine in 10 cigarette users in India consumed less than 10 cigarettes per day, yielding a low score on the HSI scale for most of the daily cigarette users. The majority of daily cigarette smokers scored ≤ 1 (low dependence) on the HSI scale both in GATS-1 and GATS-2, irrespective of their exclusive cigarette use status. The absolute value and the 95% confidence limit of positive likelihood ratios (falling below and above one) suggest that the predictability of low dependence on quit attempts and quit intention in the Indian setting is limited.

Conclusions

The utility of the HSI scale in assessing nicotine dependence among cigarette users in India is limited. This may be attributed to low average cigarette consumption, concurrent use of various tobacco products, and the sociocultural milieu of Indian smokers. This highlights the need for a new rapid nicotine dependence scale tailored to the specific patterns of tobacco use behavior prevalent in the Indian context.

## Introduction

In community settings and in settings where time is a constraint to assess nicotine dependence among tobacco users, a rapid assessment scale, the heaviness of smoking index (HSI), plays an important role. The HSI is a short version (items) of the Fagerström Test for Nicotine Dependence (FTND - six items) and the Fagerström Tolerance Questionnaire (FTQ - eight items) [1] and consists of the time to first cigarette (TTFC) and the number of cigarettes (cigarette per day [CPD]) smoked daily [2]. These two items clearly suggest that the original HSI, FTND, and FTQ were meant to assess nicotine dependence among cigarette users only.

The HSI scale measures dependence level on a seven-point scale ranging from zero to six. Daily cigarette smokers, smoking < 10 cigarettes, 11-20 cigarettes, 21-30 cigarettes, and more than 30 cigarettes are scored as zero, one, two, and three, respectively, for the item CPD. Similarly, daily cigarette smokers who smoke cigarettes within five minutes, 30 minutes, 60 minutes, and after 60 minutes of waking up are scored as three, two, one, and zero, respectively [3]. Thus, the two-item HSI scale has a minimum possible score of zero and a maximum score of six. The seven-point scale further classifies the daily cigarette smokers into low, medium or moderate, and high nicotine-dependent users. The HSI alone, or as a part of FTND, is very frequently used in clinical and community settings for the treatment and assessment of nicotine dependence purposes [4,5].

However, the scale has been in question for the product-dependence nature of tobacco dependence leading to the replacement of the term "nicotine" with "cigarette" in the FTND and renamed as the Fagerström Test for Cigarette Dependence (FTCD) [6]. Other issues that raised concerns about HSI were low average CPD settings misclassifying moderate dependence into low dependence, non-applicability in less-than-daily use settings, and simultaneous use of other tobacco product settings [7-9]. The performance of FTND and HSI in assessing dependence among light smokers (CPD < 12) is limited [10]; therefore, the application of FTND and HSI in Indian settings will remain a concern as the average CPD is less than 10 and many Indian smokers are concurrent users of other smoking and smokeless tobacco products [7,9].

Despite these concerns, HSI is frequently used in India [5,11]. In this context, the study explores the utility of HSI as a nicotine dependence measure in Indian settings by assessing its predictability to dependence outcome measures such as "quit attempt" and "quit intention" among current daily manufactured cigarette users.

## Materials and methods

The study employed an analytical study design to predict quit attempts and quit intentions based on an HSI-based dependence diagnostic tool. A subset of GATS India data (GATS-1 of 2009-10 and GATS-2 of 2016-17) available in the public domain were analyzed [12]. This study used secondary anonymous data available in the public domain. The study does not involve direct interaction with humans, so an institutional ethics committee review was not sought.

India conducts the Global Adult Tobacco Surveys (GATS) to monitor tobacco use. The GATS uses an internationally comparable questionnaire and also collects information on CPD among daily users and time to first smoke (TTFS, equivalent to TTFC as in cigarette use) considering non-cigarette smoking product use, under the smoking tobacco section (Section B) [13]. The GATS also collects information on tobacco dependence outcome measures such as quit attempts in the past year and future quit intention among current tobacco users under the section "tobacco cessation" (Section D). This indicator can be used to assess nicotine dependence using HSI among cigarette and other tobacco users. Both the quit attempts and quit intentions are well correlated with dependence level [14] and are considered dependence outcome measures.

The HSI was estimated using CPD (Item B06a: “On average, how many of the following products do you currently smoke each day? Manufactured cigarettes?”) and TTFS (Item B07: “How soon after you wake up do you usually have your first smoke?”) from the GATS data. In this study, cigarette means manufactured cigarettes only. The HSI score was converted into low (HSI score: 0-1), moderate (HSI score: 2-4), and high dependence (HSI score: 5-6) based on literature [3] and was further categorized into low (0-1) and moderate to high (2-6) dependence, considering limited respondents in moderate and high dependence category identified during analysis.

The dependence outcome measure "quit attempt" was derived from item D01: “During the past 12 months, have you tried to stop smoking?” and "quit intention" from item D08: “Which of the following best describes your thinking about quitting smoking?”. The respondents intending to quit within the next month or one year were considered as having definite quit intention and those respondents that quit some day or were not interested in quitting were categorized as having no definite quit intention.

The performance of HSI was assessed by estimating the predictability of dependence level on past quit attempts and definite quit intention using likelihood ratios [15]. In this analysis, HSI was considered a nicotine dependence diagnostic test, and low dependence predicts quit attempts and quit intention. Likelihood ratio (LR+ and LR-), a statistical parameter, can assess the effectiveness of diagnostic tests (here HSI scale). The LRs can be applied to diagnostic tests with two or more potential outcomes as well as for tests that produce continuous data [16]. Initially, Statistical Package for the Social Sciences (SPSS) v.28.0 (IBM Corp., Armonk, NY) was used for descriptive analysis and to generate a 2 x 2 contingency table, which was later used to estimate the 95% CI of LRs using MedCalc open software (MedCalc Software Ltd., Ostend, Belgium).

Further, to address the effect of simultaneous use of nicotine-containing tobacco products (concurrent use or non-exclusive use), a subanalysis of exclusive users (no concurrent use of any other smoking or smokeless tobacco products) was carried out. In this study, cigarette means manufactured cigarette only, and analysis was limited to cigarette use only. Since this subset analysis of GATS data was used to examine the individual self-reported tobacco use behavior, the GATS weight was not used in the analysis.

## Results

About 69,296 and 74,037 adults aged 15 years and above were surveyed in the first and second waves of GATS. Among these respondents, 5.6% and 3.1% were current cigarette users in the first and second waves of GATS India surveys, respectively. In the first wave of GATS, six out of 10 daily cigarette smokers used one or more other tobacco products concurrently. Similarly, in the second wave of GATS, half of the current cigarette smokers used other tobacco products concurrently. Almost all of the less-than-daily cigarette users in both the first and second waves of GATS used other tobacco products. The median CPD was five in both surveys with variation in mean CPD across gender; however, in both the first and second waves of GATS, the mean CPD was less than 10 sticks. The daily and less-than-daily smokers and their cigarettes per day consumption are given in Table 1.

**Table 1:** Manufactured cigarette users in Global Adult Tobacco Surveys (GATS) of India

Characteristics of cigarette smokers	Survey 1	Survey 2
Daily users, n (%)	3,411 (4.9)	1,878 (2.5)
Daily exclusive user, n (%)	1,369 (40.1)	944 (50.3)
Daily non-exclusive user, n (%)	2,042 (59.9)	934 (49.7)
Less-than-daily user, n (%)	510 (0.7)	427 (0.6)
Less-than-daily exclusive user, n (%)	9 (1.8)	20 (4.7)
Less-than-daily non-exclusive user, n (%)	501 (98.2)	407 (95.3)
Average daily cigarette consumption	6.71 ± 7.47	6.63 ± 6.40
Average daily cigarette consumption among males	6.80±7.58	6.62 ± 6.29
Average daily cigarette consumption among females	5.21 ± 4.98	7.01 ± 8.41
Median daily cigarette consumption	5	5

Irrespective of exclusive cigarette consumption status, nine out of 10 daily cigarette smokers smoked less than 10 cigarettes per day both in GATS-1 and GATS-2. The difference in CPD score between exclusive and non-exclusive users was statistically non-significant in both the GATS-1 and GATS-2 surveys. A higher proportion of exclusive users smoked their first cigarette within 30 minutes of waking up than their counterparts in both GATS-1 (48.9% vs 42.2%) and GATS-2 (52.2% vs 47.2%) surveys. The difference in TTFS scoring between exclusive and non-exclusive users was statistically significant in the GATS-1 and non-significant in the GATS-2 survey.

Less than half of the smokers irrespective of their exclusive cigarette use status scored low (HSI ≤ 1) in both GATS-1 and GATS-2 surveys. However, a higher proportion of exclusive smokers scored ≤1 (low dependent) on the HSI scale in both GATS-1 (46.9% vs 40.9%) and GATS-2 (48.9% vs 45%) than their non-exclusive smoking counterparts. A higher proportion of the non-exclusive cigarette smokers scored 2-6 (moderate-high dependent) than exclusive users in both GATS-1 (58.9% vs 52.2%) and GATS-2 (54.9% vs 49.1%) surveys. The difference in low, moderate, and high dependence levels between exclusive and non-exclusive cigarette smokers was statistically significant in both GATS-1 and GATS-2 surveys (Table 2).

**Table 2:** Distribution of cigarette per day (CPD), time to first cigarette (TTFC), and heaviness of smoking index (HSI) scores in Global Adult Tobacco Surveys (GATS) of India

Scoring on the HSI scale		Concurrent use of cigarettes and other tobacco products in GATS-1					Concurrent use of cigarettes and other tobacco products in GATS-2				
		Yes		No			Yes		No		
		n	%	n	%	p-value (χ^2^ test)	n	%	n	%	p-value (χ^2^ test)
Cigarette per day (score)	01-10 (0)	3041	89.2	1229	89.2	0.485	1704	90.7	857	88.9	0.974
	11-20 (1)	298	8.7	118	8.6		126	6.7	61	6.3	
	21-30 (2)	50	1.5	18	1.3		31	1.7	16	1.7	
	>30 (3)	22	0.6	4	0.3		17	0.9	10	1.0	
Time to first cigarette (score)	<5 min (3)	779	22.8	395	28.7	<0.001	491	26.1	278	28.8	0.092
	6-30 min (2)	663	19.4	277	20.1		397	21.1	226	23.4	
	31-60 min (1)	1371	40.2	539	39.1		706	37.6	333	34.5	
	>60 min (0)	588	17.2	163	11.8		283	15.1	127	13.2	
Heaviness of smoking index score	0	715	21.0	353	25.6	<0.001	468	24.9	260	27.0	0.038
	1	678	19.9	294	21.3		378	20.1	211	21.9	
	2	1260	36.9	505	36.6		670	35.7	312	32.4	
	3	597	17.5	173	12.6		289	15.4	126	13.1	
	4	118	3.5	34	2.5		53	2.8	23	2.4	
	5	26	0.8	6	0.4		14	0.7	8	0.8	
	6	7	0.2	1	0.1		5	0.3	4	0.4	
Dependence level (HSI score)	Low (0-1)	1393	40.9	647	46.9	<0.001	846	45	471	48.9	.0366
	Medium (2-4)	1975	57.9	712	51.7		1012	53.9	461	47.9	
	High (5-6)	33	1	7	0.5		19	1	12	1.2	

Table 3 illustrates the LRs for HSI's low dependence on predicting quit attempts and quit intentions. The positive likelihood of low dependence predicting quit attempts was above one (LR+ >1) among exclusive users in both GATS-1 and GATS-2 and non-exclusive users in GATS-2 only. In other instances, LR+ was below one. In all cases, the 95% CI of LR+ included one, except low dependence predicting quit attempt (LR+ = 1.25; 95% CI: 1.12-1.40) among exclusive cigarette users in the first wave of GATS. Similarly, LR- was either one, less than one, or greater than one irrespective of exclusive cigarette use status in both GATS-1 and GATS-2 surveys, with its 95% CI including one, except for low dependence predicting quit attempt among exclusive users in GATS-2 (LR- = 1.15; 95% CI: 1.01-1.30). The LR+ was less than or greater than one, with its 95% CI including one, for predicting definite quit intention among exclusive or non-exclusive users in both the GATS surveys.

**Table 3:** Predictability of low dependence on quit attempt and quit intention GATS: Global Adult Tobacco Survey; HSI: Heaviness of Smoking Index; LR: Likelihood ratio, CI: Confidence interval; LCI: Lower CI; UCI: Upper CI.

Survey	Exclusive cigarette use	HSI dependence level	Past quit attempt						Definite quit intention					
			Yes		No		LR	95% CI of LR	Yes		No		LR	95% CI of LR
			n	%	n	%		LCI-UCI	n	%	n	%		LCI-UCI
GATS-1 (2009)	Yes	Low	245	54.8	398	43.9	1.25	1.12-1.40	158	50.6	399	45	1.13	0.99-1.28
		Mod-High	202	45.2	508	56.1	0.81	0.72-0.91	154	49.4	488	55	0.9	0.79-1.02
GATS-2 (2016)	Yes	Low	144	45.3	327	52.2	0.87	0.75-1.00	76	46.6	380	50.8	0.92	0.77-1.10
		Mod-High	174	54.7	299	47.8	1.15	1.01-1.30	87	53.4	368	49.2	1.08	0.92-1.27
GATS-1 (2009)	No	Low	203	35.7	533	36.9	0.97	0.85-1.10	124	33.4	463	36.4	0.92	0.78-1.08
		Mod-High	365	64.3	913	63.1	1.02	0.95-1.09	247	66.6	808	63.6	1.05	0.96-1.14
GATS-2 (2016)	No	Low	129	41.7	246	39.5	1.06	0.90-1.25	59	39.9	292	39.7	1	0.81-1.25
		Mod-High	180	58.3	377	60.5	0.96	0.86-1.08	89	60.1	444	60.3	1	0.86-1.15

## Discussion

About 90% of the cigarette users smoked less than 10 cigarettes per day, yielding an effective score of "0" on the HSI scale for most of the users. Close to half of the cigarette smokers scored ≤1 (low dependence) on the HSI scale in both GATS-1 and GATS-2. Earlier studies have reported that Indian smokers scored low on the HSI scale because of lower mean cigarette consumption per day [7]. Since HSI assesses nicotine dependence, intake of nicotine from concurrent use of other tobacco products may affect both CPD and TTFS scoring in Indian settings and has the potential to lower the HSI scale [9] and hence the dependence level. Another important factor in nicotine dependence on tobacco products is the nicotine content of tobacco products consumed. The psychoactive substance nicotine can be sourced from only one product (exclusive use) and concurrent use of other tobacco products (non-exclusive use). The quantity of tobacco products and frequency of use (morning or other time) can be influenced by nicotine content per stick [17]. As Indian consumers consume a myriad variety of tobacco products concurrently, the HSI scale scoring and classification might systematically deviate toward lower dependence. This bias needs researchers’ attention while recommending HSI use in Indian settings.

There was a statistically significant difference in dependence level between exclusive and non-exclusive users. A higher proportion of exclusive smokers scored low dependence than their non-exclusive smoking pattern. The concurrent use of other smoking or smokeless tobacco products may affect the time to the first cigarette, while the CPD scoring may remain the same due to overall low CPD consumption. Therefore, ideally, non-exclusive uses are supposed to be scored lower than the exclusive cigarette users on the existing HSI scale; however, the opposite finding indicates limited utility of the HSI scale in multiple tobacco product use settings. Indian researchers previously questioned the imprecise definition of current use, former use, and quit attempts in the GATS survey, resulting in 21.2% invalid responses in the GATS-1 survey [18]. Therefore, the GATS data used might have influenced the result to some extent.

Traditionally, LRs are used in assessing diagnostic test performance. In this study, HSI is considered a dependence diagnostic test, and low dependence is expected to be associated with higher quit attempts and higher definite quit intentions. The absolute value and confidence limits of likelihood ratios suggest that the utility of HSI as a dependence measure in Indian settings is very limited. The utility of FTND and HSI in settings where CPD < 12 is very limited [10]. The short HSI can be an effective screening tool to detect nicotine dependence among daily smokers with higher dependence levels. However, the FTND is considered to be a more accurate measure for groups or subpopulations with low levels of nicotine addiction, such as women [19]. This study's results also reaffirm a similar view.

In this study, a subanalysis for exclusive cigarette smokers does not yield a higher utility of the HSI scale in predicting quit attempt and quit intention. The nicotine dependence culture is specific [6]. Nicotine dependence also varies with the genetic makeup of the population [20]. Thus, the suitability of HSI in the Indian population may be inherently different from other populations with higher mean CPD consumption as cigarette is the major tobacco product.

Limitations

This study used cross-sectional data limiting the assessment of temporality between the dependence level and its outcome measure. The data in the GATS survey is self-reported and thus carries an inherent risk of self-reporting data. The survey did not use any other dependence scale, limiting the assessment of the criterion validity of the HSI dependence scale. As the LR varies with prevalence, sensitivity, specificity, and spectrum of dependence in the population [16], the result needs careful interpretation.

## Conclusions

The HSI scale is a subset of FTND or FTCD and has only two items such as CPD and TTFS. Hence, it is expected to be beneficial in the rapid assessment of nicotine dependence in busy clinical settings and usage by frontline health workers in community settings. However, the HSI scale and FTND are yet to be validated in the Indian context. The HSI scale yielded systematically low scores in this study owing to lower CPD use and concurrent use of other tobacco products. There may be a role of culture, purchasing power, and genetic makeup of the Indian population. In this context, a modified scale with a different scoring pattern for the Indian population is essential. The modified scoring pattern should give due weightage to the sociocultural milieu of Indian tobacco users. Considering population specific nature of nicotine dependence, any tool to be used as an estimator of nicotine dependence needs to be validated in the Indian context, before recommending its use. Further contextual research using biological validation methods is essential to uphold the scientific utility of nicotine dependence scales.
